# Supplementary material for: Investigating the mediating role of emotional intelligence in the relationship between internet addiction and mental health among university students
Source: PLOS Digit Health. 2024 Nov 20;3(11):e0000639. doi: 10.1371/journal.pdig.0000639 (PMC11578529; doi:10.1371/journal.pdig.0000639)
Supplement: Attachment — Submitted filename: Response to the Editors.docx [file pdig.0000639.s002.docx]

PDIG-D-24-00085R1

Investigating the Mediating Role of Emotional Intelligence in the Relationship between Internet Addiction and Mental Health among University Students

Dear Dr. Zewude,

Thank you for submitting your manuscript entitled "Investigating the Mediating Role of Emotional Intelligence in the Relationship between Internet Addiction and Mental Health among University Students" to PLOS Digital Health. Your manuscript files have been checked in-house, but before we can proceed we need you to address the following issues:

1. Please provide separate figure files in .tif or .eps format only and remove any figures embedded in your manuscript file. Please also ensure that all files are under our size limit of 10MB. For more information about figure files please see our guidelines: <https://journals.plos.org/digitalhealth/s/figures> <https://journals.plos.org/digitalhealth/s/figures#loc-file-requirement>
2. Please provide an Author Summary. This should appear in your manuscript between the Abstract (if applicable) and the Introduction, and should be 150–200 words long. The aim should be to make your findings accessible to a wide audience that includes both scientists and non-scientists. Sample summaries can be found on our website under Submission Guidelines: https://journals.plos.org/digitalhealth/s/submission-guidelines#loc-parts-of-a-submission
3. In the online submission form, you indicated that "The corresponding authors hold the data sets generated and analyzed during the study and are willing to share them upon request". All PLOS journals now require all data underlying the findings described in their manuscript to be freely available to other researchers, either 1. In a public repository, 2. Within the manuscript itself, or 3. Uploaded as supplementary information.

This policy applies to all data except where public deposition would breach compliance with the protocol approved by your research ethics board. If your data cannot be made publicly available for ethical or legal reasons (e.g., public availability would compromise patient privacy), please explain your reasons by return email and your exemption request will be escalated to the editor for approval. Your exemption request will be handled independently and will not hold up the peer review process, but will need to be resolved should your manuscript be accepted for publication. One of the Editorial team will then be in touch if there are any issues.

Your manuscript has been returned to your account. Please log on to PLOS Editorial Manager at https://www.editorialmanager.com/pdig/ to access your manuscript.

Your manuscript can be found in the "Revisions Sent Back to the Author" link under the New Submissions menu. After you have made the changes requested above, please be sure to view and approve the revised PDF after rebuilding the PDF to complete the resubmission process.

Please note that these changes have been requested to comply with journal guidelines and your revised manuscript will not be sent to review until you have fully adhered to our requests. Once your paper has been seen by an Editor we may return it to you for further information or amendments.

Please resolve these issues and resubmit your manuscript within 30 days. If you need more time, please email the journal office at digitalhealth@plos.org. We are happy to grant extensions of up to one month past this due date. If we do not hear from you within 30 days, we will withdraw your manuscript on your behalf.

Thank you for submitting your work to PLOS Digital Health.

**Response to the Editors**

*Thank you for reviewing our manuscript titled "Investigating the Mediating Role of Emotional Intelligence in the Relationship between Internet Addiction and Mental Health among University Students." We appreciate the feedback and have addressed the issues raised. Below are our responses to each point:*

*1. We apologize for the oversight. We have now provided separate figure files in .tif format and removed any embedded figures in the manuscript file. All figure files are within the size limit of 10MB. We have followed the figure file requirements outlined in the guidelines provided.*

*2. Thank you for reminding us to include an Author Summary. We have added an Author Summary section between the Abstract and the Introduction of the manuscript.*

**Authors Summary**

This study examined the psychometric properties and relationships between internet addiction, emotional intelligence, and mental health among Ethiopian university students. The measures used - the Wong Law Emotional Intelligence Scale (WLEIS-S), Internet Addiction Scale (IAS), and Keyes' Mental Health Continuum-Short Form (MHC-SF) - were found to be reliable and valid in the Ethiopian context. The findings indicate that higher levels of internet addiction are associated with lower emotional intelligence and poorer mental health. Conversely, emotional intelligence was positively correlated with mental health. The study determined that internet addiction negatively predicts both emotional intelligence and mental health, while internet craving directly predicts emotional intelligence and indirectly and negatively predicts mental health through emotional intelligence. These results highlight the detrimental effects of excessive internet use on the mental well-being of university students. The authors recommend interventions to raise awareness, promote healthy digital habits, and enhance emotional intelligence as a means of supporting students' mental health and preventing internet addiction.

*3. Regarding the data sharing policy, we acknowledge the requirement for all data underlying our findings to be freely available to other researchers. We are committed to promoting transparency and open access to research. Therefore, we kindly aatched in supplementary files with the article.*

*We included: All data underlying the findings described in this manuscript should be made freely available to other researchers. The data should be uploaded as supplementary material to accompany the analysis.*
